# Differential retention of adalimumab and etanercept biosimilars compared to originator treatments: Results of a retrospective French multicenter study

**DOI:** 10.3389/fmed.2022.989514

**Published:** 2022-10-06

**Authors:** Guillaume Larid, Guy Baudens, Alexis Dandurand, Pascal Coquerelle, Vincent Goeb, Marie Hélène Guyot, Laurent Marguerie, Frédéric Maury, Eric Veillard, Eric Houvenagel, Jean-Hugues Salmon, René-Marc Flipo, Elisabeth Gervais

**Affiliations:** ^1^Department of Rheumatology, CHU Poitiers, Poitiers, France; ^2^LITEC Laboratory, University of Poitiers, Poitiers, France; ^3^Private Practice, Valenciennes, France; ^4^Department of Rheumatology, Bethune Hospital Center, Bethune, France; ^5^Department of Rheumatology, Hôpital Nord, University Hospital of Amiens-Picardie, Amiens, France; ^6^Department of Rheumatology, Hôpital Victor Provo, Hospital of Roubaix, Roubaix, France; ^7^Department of Rheumatology, Institut François Calot, Berck-Sur-Mer, France; ^8^Private Practice, Beuvry, France; ^9^Private Practice, Saint-Malo, France; ^10^Department of Rheumatology, Hôpital Saint Philibert, Hospital of Lomme, Lomme, France; ^11^Department of Rheumatology, Hôpital Maison Blanche, Reims University Hospital, CEDEX, Reims, France; ^12^Department of Rheumatology, CHU Lille, Lille, France

**Keywords:** TNF-inhibitors, biosimilars, survival, retention rate, predictive factors

## Abstract

**Objectives:**

Previous studies demonstrated equivalence in terms of efficacy and safety of biosimilars (bsDMARDs) compared to original treatments (boDMARDs) and in switching situations. Less is known about what happens when initiating a bsDMARD in a molecule naïve patient. The objectives of our study were to compare the retention of treatment of subcutaneous boDMARDs and bsDMARDs globally, depending on the disease [rheumatoid arthritis (RA), spondyloarthritis (SpA), or psoriatic arthritis (PsA)], molecule [etanercept (ETN) or adalimumab (ADA)], line of treatment, or presence of citrate in the context of first use of each molecule (namely initiation) and to analyze treatment retention’s predictive factors.

**Materials and methods:**

This multicenter retrospective study used data from shared medical records of the RIC-FRANCE network, encompassing the prescription of hospital rheumatologists and attached practitioners, of patients with RA, SpA, or PsA, with the starting ETN between 03/10/2016 and 31/07/2020, or ADA between 23/10/2018 and 31/07/2020. Clinical data were collected from medical records. Retention analysis was performed using Kaplan–Meier curves and the log-rank test. Retention’s predictive factors were analyzed using Cox proportional-hazard ratio.

**Results:**

Eight hundred forty-five prescriptions were analyzed: 340 boDMARDs and 505 bsDMARDs. About 57% of prescriptions concerned women. The mean age was 51.8 years. About 38% were prescriptions for RA, 16% for PsA, and 46% for SpA. An increase in the initiation over time was observed for both ETN and ADA. The retention rate of bsDMARDs was superior to boDMARDs’ one (39 vs. 23 months; *p* = 0.045). When molecules are compared, the difference was significant only for ETN (45 vs. 19 months for boDMARD; *p* = 0.0265). When comparing diseases, the difference in favor of bsDMARDs was significant in patients with RA only (*p* = 0.041). Citrated treatments displayed better retention compared to citrate-free treatments (*p* = 0.0137). Multivariable analysis of predictive factors for the cessation of treatment found shorter disease duration, boDMARD prescription, hospital practitioner prescription, late line of treatment, and female sex as significant. More side effects were observed with boDMARDs, especially more infections (17.8% vs. 7.8%).

**Conclusion:**

Even if bsDMARDs’ prescription increases over time, its penetration rate is still below expectations. bsDMARDs displayed better retention compared to boDMARDs, especially for ETN, and in patients with RA. Citrated treatments had better retention. Prescription by a full-time hospital-based rheumatologist is associated with poorer retention.

## Introduction

The treatment of rheumatic diseases has been improved in the last 20 years with the advent of biological disease-modifying anti-rheumatic drugs (bDMARDs) (1–3). Those treatments are the keystones of rheumatic disease management, as shown in the management guidelines for rheumatoid arthritis (RA) (4), spondyloarthritis (SpA) (5), and psoriatic arthritis (PsA) (6).

Original bDMARDs (boDMARDs) are sold with a period of exclusivity to allow pharmaceutical companies to recoup the money spent on research and development. Even if efficient, those treatments are costly. In 2012, among the top 15 drugs, there were seven biological agents, four of which (adalimumab, etanercept, infliximab, and rituximab) were originators used in rheumatic disease treatment with total sales amounted to $32.6 billion (7, 8).

In order to reduce healthcare costs of bDMARDs, biosimilar drugs of boDMARDs (bsDMARDs) have been marketed at a reduced price compared to boDMARDs. After this period of boDMARDs’ exclusivity, bsDMARDs can be drawn to the market. According to the European Medicine Agency, biosimilar medicine is a medicine highly similar to another biological medicine already marketed (9). bsDMARDs have been proven to show non-meaningful clinical differences with respect to their reference product (10).

The regulation of biosimilar products is different between the European Union (EU) and the United States (US), which led to an earlier approval and marketing of bsDMARDs in the EU (11).

Biosimilars’ particular development is based on comprehensive comparability studies with the original biologic. Those studies have to demonstrate that the candidate medicine is highly similar, notwithstanding the natural variability inherent to its nature, and that there are no clinically meaningful differences between the biosimilar and the reference medicine in terms of safety, quality, and efficacy (9, 11).

In France, etanercept (ETN) and adalimumab (ADA) have multiple bsDMARDs available: HULIO, AMGEVITA, IMRALDI, IDACIO, and HYRIMOZ for ADA, ERELZI, and BENEPALI for ETN.

The prescription of bsDMARDs instead of boDMARDs had a dramatic impact on healthcare costs. Indeed, it has been demonstrated that biosimilars could reduce direct spending on biological drugs by $54 billion from 2017 to 2026 or by a range of $24 to $150 billion over the same period in the United States (12). To emphasize this point, it has been shown that the delay of approval for ADA biosimilars resulted in an excess of spending of $ 2.19 billion for MEDICARE in the United States (13). In France, cost savings generated by the use of biosimilars to TNF-inhibitor agents have been demonstrated to exceed €820 million over 5 years (2015–2020) (14). With the cost saving nature of biosimilars, the choice between the prescription of bsDMARDs and that of boDMARDs at initiation is still left to the physician’s own appreciation.

Even if they are described as equivalent to boDMARDs in terms of efficacy, the retention of treatment of bsDMARDs compared to boDMARDs is variable across studies with an absence of differences in some studies (15–20) or better retention for bsDMARDs (16, 21) or boDMARDs in others (22–25). The majority of the published studies focus on switching a boDMARD to a bsDMARD, but only a few focus on comparing retention rates at the initiation of treatments.

The objectives of our study were to compare the retention of treatment of boDMARDs and bsDMARDs, compare the retention of treatment depending on disease (RA, SpA, or PsA), molecule (etanercept or adalimumab), line of treatment, or presence of citrate in the context of first use of each molecule (namely initiation). Predictive factors of treatment retention, as well as the side effects of bsDMARDs and boDMARDs, have also been analyzed.

## Materials and methods

### Patients

A retrospective, observational, multicenter study was conducted using medical records from the multicenter RIC-France registry that encompasses patients followed by rheumatologists working full-time in French hospitals (namely, hospital practitioners) and rheumatologists with a predominantly office-based activity (namely, attached practitioner). This registry had already been used for clinical studies on rheumatic diseases (26). Patients are included in the database and data are filled in by their rheumatologists during consultations. In the context of this study, data of each patient were completed based on their original medical records.

The inclusion criteria were patients with rheumatoid arthritis (RA) fulfilling the 2010 ACR/EULAR classification criteria (27), axial spondyloarthritis (SpA) fulfilling the 2009 ASAS classification criteria (28), or psoriatic arthritis (PsA) fulfilling the CASPAR classification criteria (29), initiating treatment by ETN or ADA without previous use of the molecule chosen, whatever the line of treatment. For ETN, patients beginning their treatments from 3 October 2016 to 31 July 2020 were included. Patients with ADA beginning their treatments from 23 October 2018 to 31 July 2020 were included. These dates are the commercialization date of the first biosimilar of each treatment in France.

Patients previously treated with ETN or ADA and switched to a biosimilar were excluded.

### Assessments

Patients characteristics collected at initiation were as follows: age, sex, type of rheumatic disease, diagnosis date, duration between diagnosis and initiation of treatment, total number of previously received bDMARDs, concomitant treatment with methotrexate (MTX) and corticosteroids, DAS28-ESR and DAS28-CRP at initiation for RA and PsA, and ASDAS-CRP and BASDAI at initiation for SpA.

Disease activity was dichotomized into low, moderate, or high disease activity based on the usual threshold for each activity score (30, 31). Low activity was defined as DAS28 < 3.2 or ASDAS < 2.1. Moderate disease activity was defined as 3.2 < DAS28 < 5.1 or 2.1 < ASDAS-CRP < 3.5. High disease activity was defined as DAS28 > 5.1 or ASDAS-CRP > 3.5.

Reasons for the cessation of treatment were collected and classified as a side effect, primary inefficiency, secondary inefficiency, and a switch without a medical reason. Primary inefficiency was defined as an absence of response in the first 6 months following initiation of treatment. Cessation of treatment was defined as secondary inefficiency if the loss of response occurs after 6 months of initial therapeutic response.

Citrate-free treatments were those without sodium citrate and/or citric acid (HUMIRA, ENBREL, BENEPALI, HULIO, and AMGEVITA).

### Statistical analysis

Qualitative data were expressed as percentages and quantitative data as means ± standard deviations. Analysis was conducted using Student’s *t*-test (or Wilcoxon, as appropriate) for quantitative data and Chi^2^ (or Fisher exact test) for qualitative data. For three groups’ comparison of quantitative data, an ANOVA analysis using the Kruskal–Wallis test was performed in association with Dunn’s multiple comparisons test. The correlation between time and treatment prescription was analyzed using Spearman’s coefficient of correlation analysis. To compare treatment retention, a log-rank test using Kaplan–Meier curves was used. For univariable and multivariable analyses of predictive factors of retention, a Cox proportional-hazards regression was performed. All variables were included in the multivariable analysis using an enter method. A *p*-value of 0.05 was considered significant. Statistical analysis was performed using GraphPad Prism (GraphPad Software, California) and MedCalc (MedCalc Software Ltd, Belgium).

### Ethics

The study was approved by the local institutional ethics committee and conducted in accordance with the Declaration of Helsinki. Written consent was not required according to the MR-004 French legislation.

## Results

### Description of the population

Eight hundred and forty-five prescriptions fulfilled the inclusion criteria of the study and were analyzed. Detailed characteristics are shown in Table 1.

**TABLE 1:** Detailed characteristics of all 845 patients.

	All prescriptions (*n* = 845)	boDMARDs (*n* = 340)	bsDMARDs (*n* = 505)	*p*
**Demographic characteristics**				
Women, n (%)	482 (57%)	200 (58.8%)	282 (55.8%)	0.69
Men, n (%)	363 (43%)	140 (41.2%)	223 (44.2%)	
Mean age (± SD)	51.8 (± 14.54)	51.76 (± 15.25)	51.91 (± 14.06)	0.9276
**Diagnosis**				
Rheumatoid arthritis, n (%)	321 (38%)	117 (34.4%)	204 (40.4%)	0.48
Psoriatic Arthritis, n (%)	135 (16%)	56 (16.5%)	79 (15.6%)	
Axial Spondyloarthritis, n (%)	411 (46%)	167 (49.1%)	222 (44%)	
**Disease duration (mean, months)**				
Rheumatoid arthritis	106.9	92.23	115.5	0.146
Psoriatic Arthritis	115.4	122.6	110.3	0.620
Axial Spondyloarthritis	98.23	82.97*	110.2*	*: 0.016
Total	104.4	92.67*	112.5*	*: 0.012
**Associated treatments at initiation^1^**				
Corticosteroids, n (%)	115 (25.2%)	53 (30.6%)	62 (21.9%)	0.114
Methotrexate, n (%)	215 (47.1%)	79 (45.7%)	136 (48.1%)	0.603
All csDMARDs and/or corticosteroids, n (%)	335 (73.5%)	132 (76.3%)	203 (71.7%)	0.563
**Previous bDMARDs, n (%)**				
0	474 (56.1%)	181 (53.2%)	293 (58%)	0.100
1	235 (26.3%)	86 (25.3%)	136 (26.9%)	
≥2	149 (17%)	73 (21.5%)	71 (14.1%)	
Mean previous bDMARDs	0.706	0.792	0.648	0.107
**Molecule, n (%)**				
Adalimumab	290 (34.3%)	121 (35.6%)	169 (33.5%)	0.816
Etanercept	555 (65.7%)	219 (64.4%)	336 (66.5%)	
Citrated treatment	97 (11.5%)	0 (0%)	97 (19.2%)	–
Non-citrated treatment	748 (88.5%)	340 (100%)	408 (80.8%)	
**Disease activity at initiation^2^**	*n* = 269	*n* = 174	*n* = 95	
Low, n (%)	61 (22.7%)	40 (23%)	21 (22.1%)	0.3457
Moderate, n (%)	145 (53.9%)	98 (56.3%)	47 (49.5%)	
Severe, n (%)	63 (23.4%)	36 (20.7%)	27 (28.4%)	
**Place of initiation, n (%)**				
Center N°1 CH	45 (5.3%)	2 (4.4%)	43 (95.6%)	**<0.0001**
Center N°2 CH	148 (17.5%)	20 (13.5%)	128 (86.5%)	**<0.0001**
Center N°3 CHU	34 (4%)	8 (23.5%)	26 (76.5%)	**<0.0001**
Center N°4 CHU	59 (7%)	16 (27.1%)	43 (72.9%)	**<0.0001**
Center N°5 CHU	223 (26.4%)	99 (44.4%)	124 (55.6%)	**0.018**
Center N°6 CH	125 (14.8%)	61 (48.8%)	64 (51.2%)	0.704
Center N°7 CHU	106 (12.6%)	60 (56.6%)	46 (43.4%)	0.054
Center N°8 CH	52 (6.1%)	39 (75%)	13 (25%)	**<0.0001**
**Hospital rheumatologist**	792 (93.7%)	305 (89.7%)	487 (96.4%)	**<0.0001**
Center N°9 - Attached practitionners	53 (6.3%)	35 (66.0%)	18 (33.4%)	

^1^Only patients with RA and PsA; ^2^low disease activity was defined as DAS28-ESR < 3.2 for patients with RA and PsA and ASDAS < 2.1 for patients with SpA; moderate disease activity was defined as 3.2 ≤ DAS28-ESR < 5.1 or 2.1 ≤ ASDAS < 3.5; high disease activity was defined as DAS28-ESR ≥ 5.1 or ASDAS ≥ 3.5; in the green prescription of bsDMARDs > boDMARDs, in the red-green prescription of bsDMARDs < boDMARDs, in orange, no difference between the prescription of bsDMARDs and boDMARDs.

* Compared values.

About 57% of prescriptions concerned women. The mean age at initiation was 51.8 years. Of the 845 patients, 38% of them were patients with RA, 16% of them were patients with PsA, and 46% of them were patients with SpA. Among RA and PsA prescriptions, 47.1% of patients had methotrexate associated with bDMARDs and 25.2% of patients were under corticosteroid therapy.

Three hundred forty prescriptions were of boDMARD treatment, including 121 prescriptions of ADA and 219 of ETN, and 505 prescriptions were of bsDMARD treatment, including 336 prescriptions of ETN (BENEPALI^®^ = 278 prescriptions; ERELZI^®^ = 58 prescriptions) and 169 of ADA (AMGEVITA^®^ = 106 prescriptions; HULIO^®^ = 24 prescriptions; IMRALDI^®^ = 23 prescriptions; IDACIO^®^ = 11 prescriptions; and HYRIMOZ^®^ = 5 prescriptions).

The comparison of the bsDMARD and boDMARD groups showed higher disease duration in patients with SpA in the bsDMARD group (110.2 months vs. 82.97 months; *p* = 0.016). The majority of the hospital centre (5/8) prescribed significantly more bsDMARD than boDMARDs, while two of them equally prescribed both categories of treatment. Attached practitioners prescribed more boDMARDs.

### Time trends of biosimilar drugs of original biological disease-modifying rheumatic drugs prescription

**FIGURE 1:**
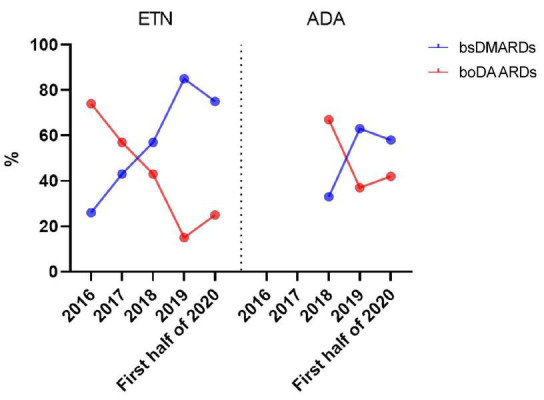
Evolution of bsDMARD and boDMARD prescriptions over time.

An evolution of bDMARDs prescription over time is observed with an increase of bsDMARD prescription for both ETN and ADA: over 4 years for ETN and 2 years for ADA (Figure 1).

A significant increase of biosimilar ETN prescription over time was observed (Rho = 0.900; *p* = 0.0374) but not for ADA (Rho = 0.500; *P* = 0.6667).

### Retention of treatment analysis

#### Retention analysis of original biological disease-modifying anti-rheumatic drugs vs. biosimilar drugs of biological disease-modifying anti-rheumatic drugs

**FIGURE 2:**
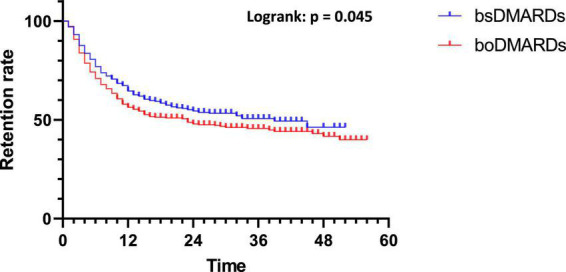
Retention of bsDMARDs vs. boDMARDs.

First, the comparison of boDMARDs vs. bsDMARDs’ retention was analyzed (Figure 2). The median retention length of bsDMARDs was 39 months, while that of boDMARDs one was 23 months (*p* = 0.045).

**FIGURE 3:**
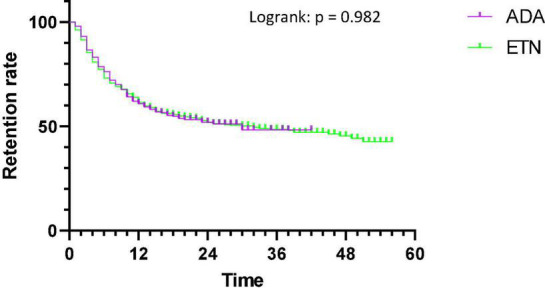
Retention of ADA and ETN.

While looking at retention differences between molecules, there were no differences in retention between ADA and ETN (*p* = 0.982) with a median retention length of 30 months for ADA and 32 months for ETN (Figure 3).

**FIGURE 4:**
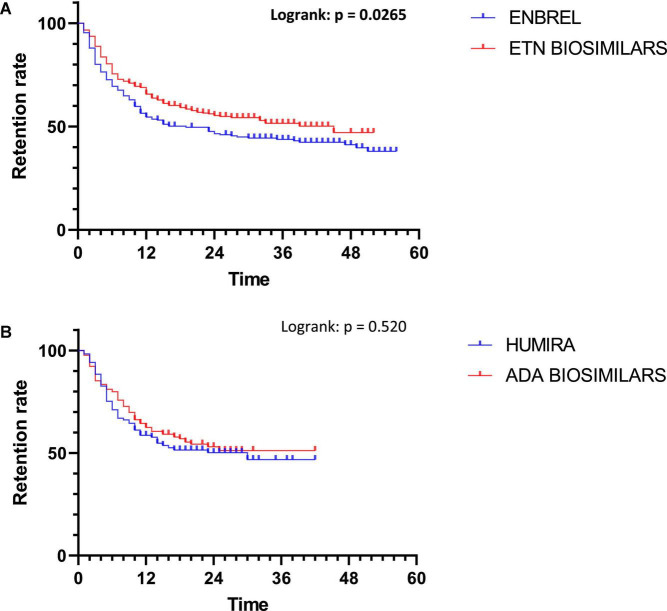
Comparison of bsDMARD and boDMARD retention for ETN **(A)** and ADA **(B)**.

For ETN, biosimilars’ retention was longer than ENBREL’s one with a median length of 45 months for biosimilars vs. 19 months for ENBREL (*p* = 0.0265) (Figure 4A).

For ADA, there were no differences in retention between groups (*p* = 0.520). The median retention length was 30 months for HUMIRA, but it was not calculable for biosimilars (Figure 4B).

#### Retention analysis depending on the line of treatment

**FIGURE 5:**
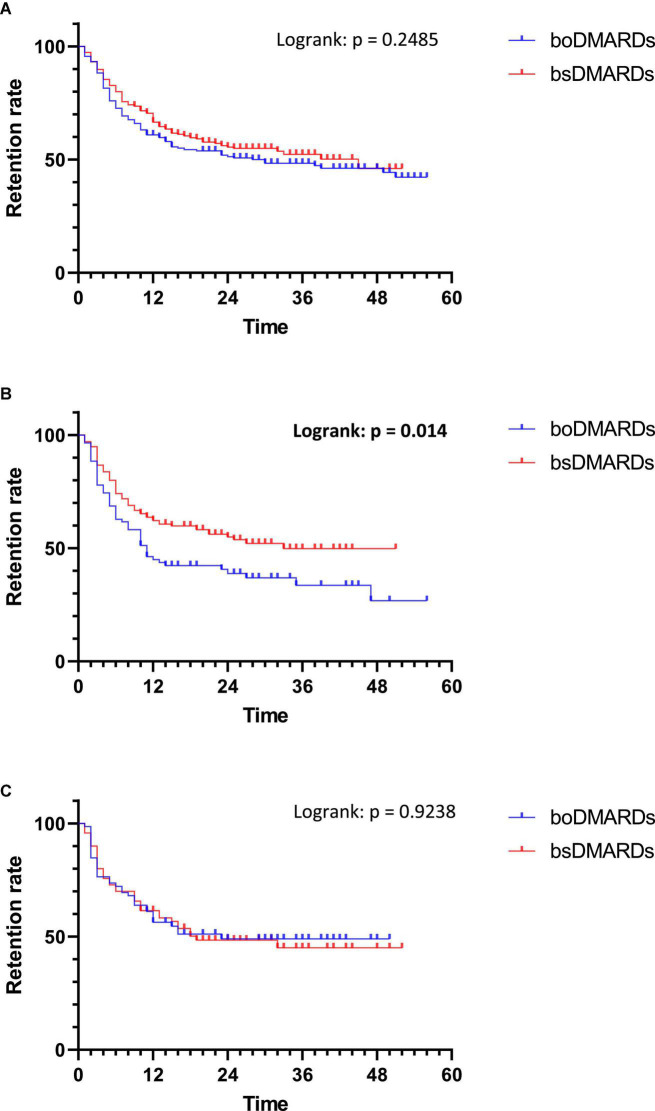
Retention of treatments depending on the line of treatments. **(A)** First line of treatment; **(B)** Second line of treatment; **(C)** Third line of treatment.

When prescribed as first-line bDMARDs, there were no differences between boDMARDs’ and bsDMARDs’ retention (*p* = 0.2485), with a median retention length of 30 months and 45 months, respectively (Figure 5).

When prescribed as second-line bDMARDs, bsDMARDs’ retention was higher than boDMARDs’ (*p* = 0.014), with a median retention length of 33 months and 11 months, respectively.

When prescribed as third-line bDMARDs and more, there were no differences between boDMARDs’ and bsDMARDs’ retention (*p* = 0.9238), with a median retention length of 23 months and 19 months, respectively.

#### Retention analysis depending on the disease

**FIGURE 6:**
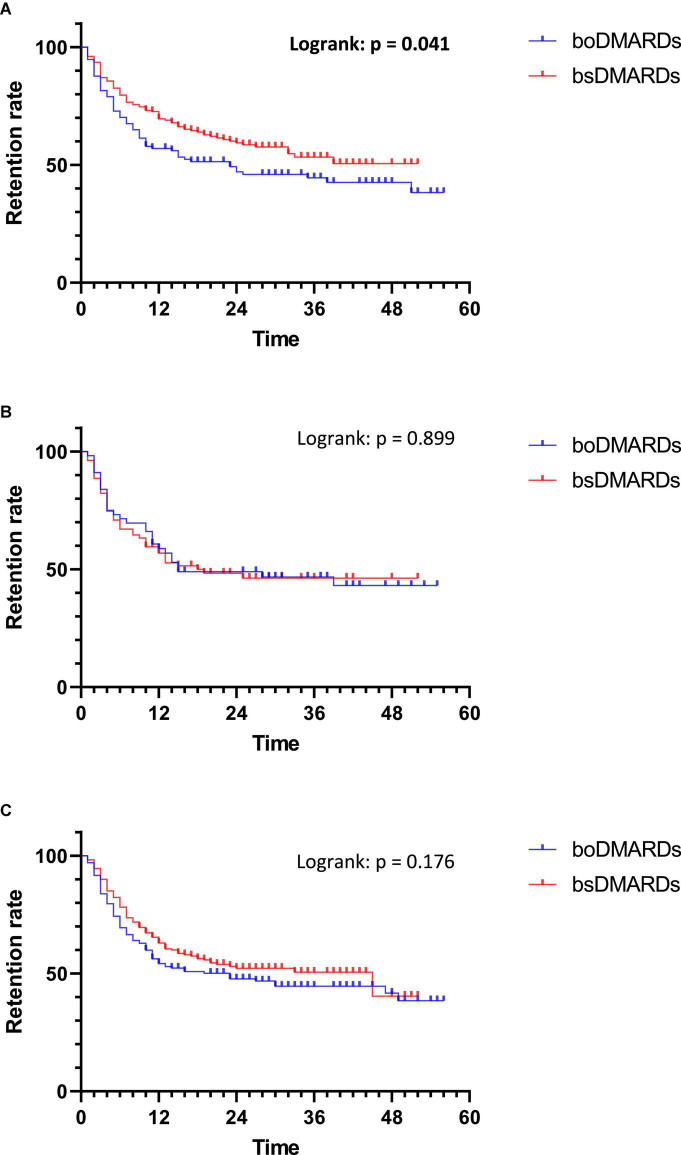
Retention of treatments in each rheumatic disease. **(A)** Rheumatoid arthritis; **(B)** Psoriatic arthritis; **(C)** Spondyloarthritis.

In patients with RA, bsDMARDs’ retention was longer than boDMARDs’ one (*p* = 0.041). The median retention length of boDMARDs was 23 months, while it was not calculable for bsDMARDs because it never falls under 50% (Figure 6).

No differences of retention were observed in patients with PsA and SpA (*p* = 0.899 and *p* = 0.176, respectively).

#### Retention analysis depending on citrate presence in treatments

**FIGURE 7:**
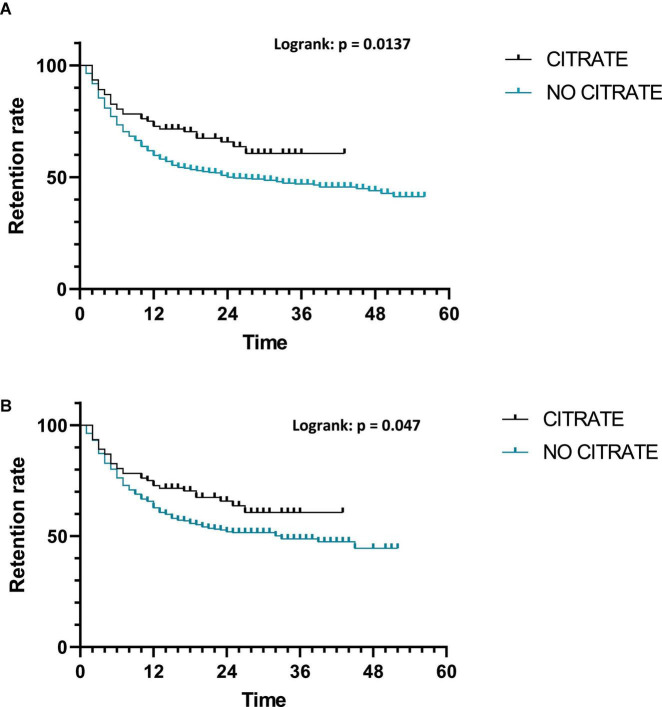
Retention of treatments depending on citrate presence. **(A)** All treatments; **(B)** bsDMARDs only.

The comparison of retention length between citrated bsDMARDs and non-citrated bsDMARDs showed higher retention in citrated bsDMARDs (*p* = 0.047) with a median retention length of 33 months for non-citrated treatments. The retention length was not calculable for citrated bsDMARDs (Figure 7).

### Analysis of predictive factors of retention

Predictive factors of the cessation of treatment were analyzed.

Concerning the retention of treatments for all ADA and ETN (biosimilars and originators) (Table 2), older age, long disease duration, bsDMARDs, citrate presence, attached practitioner prescription, early line of treatment, and male sex were predictive factors of treatment retention in the univariable analysis. Disease duration, bsDMARDs, attached practitioner prescription, early line of treatment, and male sex remained significant factors in the multivariable analysis.

**TABLE 2:** Predictive factors of cessation of treatment in univariable and multivariable analyses.

	Univariable analysis			Multivariable analysis		
	*p*	Hazard ratio	CI 95%	*p*	Hazard ratio	CI 95%
Age	**0.0166**	**0.9920**	**0.9856–0.9985**	0.4838	0.9972	0.9895–1.0050
Disease duration	**0.0001**	**0.9970**	**0.9965–0.9989**	**0.0003**	**0.9976**	**0.9963–0.9989**
bsDMARDs	**0.0365**	**0.8133**	**0.6701–0.9871**	**0.0370**	**0.7903**	**0.6335–0.9859**
Citrate presence	**0.0121**	**0.6301**	**0.4391–0.9040**	0.1622	0.7460	0.4946–1.1251
RA	–	1.00	REF	–	1.00	REF
PsA	0.2801	1.1678	0.8813–1.5476	0.6305	1.0838	0.7809–1.5042
SpA	0.4661	1.0821	0.8753–1.3377	0.6914	0.9434	0.7076–1.2578
Hospital practitioner	**0.0059**	**2.0208**	**1.2249–3.3340**	**0.0074**	**2.0553**	**1.2130–3.4825**
Line of treatment	**0.0074**	**1.1365**	**1.0350–1.2481**	**0.0008**	**1.1933**	**1.0757–1.3238**
ADA	–	1.00	REF	–	1.00	REF
ETN	0.7900	1.0285	0.8364–1.2647	0.9268	0.9890	0.7805–1.2531
Methotrexate coprescription	0.1217	0.8367	0.6676–1.0486	0.2693	0.8564	0.6505–1.1275
Female sex	**0.0009**	**1.4012**	**1.1482–1.7100**	**0.0247**	**1.2911**	**1.0330–1.6136**

CI 95%: confidence interval 95%; bold values are significant.

While looking at bsDMARDs’ retention (Table 3), older age, long disease duration, citrate presence, early line of treatment, and male sex were predictive factors of treatment retention in the univariable analysis. Only disease duration and early line of treatment remained significant in the multivariable analysis.

**TABLE 3:** Predictive factors of bsDMARDs’ cessation in univariable and multivariable analyses.

	Univariable analysis			Multivariable analysis		
	*p*	Hazard ratio	CI 95%	*p*	Hazard ratio	CI 95%
Age	**0.0126**	**0.9887**	**0.9799–0.9976**	0.3071	0.9945	0.9841–1.0051
Disease duration	**0.0031**	**0.9977**	**0.9961–0.9992**	**0.0028**	**0.9974**	**0.9957–0.9991**
Citrate presence	**0.0385**	**0.6739**	**0.4638–0.7793**	0.2183	0.7701	0.5080–1.1673
RA	–	1.00	REF	–	1.00	REF
PsA	0.0907	1.3781	0.9504–1.9983	0.4832	1.1747	0.7490–1.8423
SpA	0.3216	1.1551	0.8686–1.5361	0.7284	0.9282	0.6095–1.4136
Hospital practitioner	0.5801	1.2368	0.5826–2.6256	0.6508	1.2101	0.5298–2.7640
Line of treatment	**0.0228**	**1.1628**	**1.0212–1.7165**	**0.0008**	**1.2827**	**1.1092–1.4834**
ADA	–	1.00	REF	–	1.00	REF
ETN	0.7601	0.9579	0.7265–1.2628	0.4609	0.8864	0.6433–1.2213
Methotrexate coprescription	0.3366	0.8656	0.6448–1.1619	0.5724	0.8955	0.6105–1.3137
Female sex	**0.0067**	**1.4450**	**1.1076–1.8852**	0.0981	1.2933	0.9536–1.7542

CI 95%: confidence interval 95%; bold values are significant.

Concerning boDMARDs’ retention (Table 4), long disease duration and attached practitioner prescription were predictive factors of treatment retention in the univariable analysis. Only attached practitioner prescriptions remained significant in the multivariable analysis.

**TABLE 4:** Predictive factors of boDMARDs’ cessation in univariable and multivariable analyses.

	Univariable analysis			Multivariable analysis		
	*p*	Hazard ratio	CI 95%	*p*	Hazard ratio	CI 95%
Age	0.3994	0.9959	0.9864–1.0017	0.9580	0.9997	0.9881–1.0114
Disease duration	**0.0334**	**0.9981**	**0.9963–0.9998**	0.0694	0.9981	0.9961–1.0001
RA	–	1.00	REF	–	1.00	REF
PsA	0.6465	0.9039	0.5870–1.3919	0.8892	0.9658	0.5923–1.5749
SpA	0.7499	0.9496	0.6910–1.3050	0.7879	0.9464	0.6338–1.4132
Hospital practitioner	**0.0016**	**2.9490**	**1.5069–5.7714**	**0.0051**	**2.6518**	**1.3399–5.2483**
Line of treatment	0.2255	1.0877	0.9494–1.2460	0.2319	1.0964	0.9428–1.2749
ADA	–	1.00	REF	–	1.00	REF
ETN	0.3930	1.1454	0.8388–1.5642	0.4885	1.1328	0.7960–1.6121
Methotrexate coprescription	0.2725	0.8205	0.5761–1.1684	0.2481	0.7845	0.5197–1.1843
Female sex	0.0598	1.3350	0.9881–1.8036	0.1282	1.2896	0.99293–1.7897

CI 95%: confidence interval 95%; bold values are significant.

### Cessation reasons and side effects

The reasons for the cessation of treatment are shown in Table 5. There were significantly more side effects for boDMARDs compared to bsDMARDs (OR = 1.571 [1.044–2.362]; *p* = 0.0320).

**TABLE 5:** Reasons for the cessation of treatment.

Cessation reason	boDMARDs	bsDMARDs	*p*	Odd ratio	CI 95%
Primary inefficiency	76 (22.4%)	89 (17.6%)	0.0890	1.345	0.9568–1.881
Side effect	51 (15%)	51 (10.1%)	**0.0320**	**1.571**	**1.044–2.362**
Secondary inefficiency	40 (11.8%)	67 (13.3%)	0.5195	0.8716	0.5802–1.325
Others	22 (6.5%)	21 (4.2%)	0.1337	1.594	0.8718–2.940
Total	190 (55.9%)	232 (45.9%)	**0.0046**	**1.491**	**1.130–1.974**

CI 95%: confidence interval 95%; bold values are significant.

The analysis of side effects showed significantly more infection in the boDMARDs group (OR = 3.406 [1.083–10.07]; *p* = 0.0440). Other side effects were not significantly different between groups (Table 6).

**TABLE 6:** Details of side effects reported.

Side effect	boDMARDs	bsDMARDs	*p*	Odd ratio	CI 95%
Cutaneous	13 (25.5%)	17 (33.3%)	0.7247	1.141	0.5646–2.300
Others	14 (27.5%)	13 (25.5%)	0.2110	1.625	0.7612–3.375
**Infection**	**9 (17.6%)**	**4 (7.8%)**	0.0440*	**3.406**	**1.083–10.07**
Digestive intolerance	5 (9.8%)	6 (11.8%)	0.7631*	1.241	0.4306–4.202
Local reaction at injection site	4 (7.8%)	6 (11.8%)	>0.9999*	0.9901	0.3131–3.673
Cancer	3 (5.9%)	2 (3.9%)	0.3968*	2.239	0.4546–12.66
Respiratory disease	2 (3.9%)	3 (5.9%)	>0.9999*	0.9901	0.1749–4.872
Uveitis	1 (2%)	1 (2%)	>0.9999*	1.487	0.07804–28.30

*Fisher’s exact test; CI 95%: confidence interval 95%; bold values are significant.

## Discussion

In this retrospective multicenter study, better retention of bsDMARDs over boDMARDs had been observed. Apart from bsDMARD prescription, identified predictive factors of retention of treatment were longer disease duration, prescription by a predominantly office-based practitioner, early line of treatment, and male sex.

One surprising result was the not-so-obvious initiation of bsDMARDs by French rheumatologists. The prescription rate of bsDMARDs was mediocre for ADA biosimilars, which culminated at 60% in 2019 while it was up to 80% for ETN in 2019. The rate is less than that indicated in recently reported results from the ART-SFR French registry focusing on the initiation of TNF-alpha inhibitors in RA, whatever the molecule. Indeed, in this study, 100% of ADA prescriptions were bsDMARDs in the second trimester of 2019, while it was superior to 90% for ETN (32). An important difference from our study is that inclusion in the ART registry is only done by hospital rheumatologists who are more prone to prescribe biosimilars. In another retrospective observational study based on the French national uniform hospital discharge data set database (PMSI), the results are more close to ours with a penetration rate of biosimilars in 74% of cases for etanercept and 77% for adalimumab (14). In this last study, ambulatory prescriptions were not taken into account, which is contrary to our study. In France, incentives to favor bsDMARD prescription are not equivalent between hospitals and are based on a financial benefit allocated to hospital departments. Moreover, those measures can be applied for only some bDMARDs and not for others. These could partly explain the disparity between centers in their prescription pattern but we did not have the information for the different centers to evaluate this point.

An inflexion of bsDMARD prescriptions was observed in the first half of 2020. We could speculate an impact of the COVID-19 pandemic outbreak, which is known to have impacted prescribing habits with hydroxychloroquine being the most cartoonish example (33). It is known that one of the most important elements in the prescription decision by physicians is their own experience with the medication (34, 35). As rheumatologists may have some fears about bsDMARDs, for some related to lack of knowledge, as highlighted by different studies (36–39), we could hypothesize that they have fallen back on treatments they have known for a longer time. Center-effect, especially between hospital-based rheumatologists and attached practitioners, observed in our study may be related to these beliefs. This is confirmed by a French study using a survey submitted to rheumatologists exploring their beliefs and knowledge (40). In the study by Jarrion et al., such a decline has been also observed for ADA but not for ETN (14). However, this last study about prescription did not differentiate initiation and switch situations, which makes it not completely comparable to this study.

The univariable analysis identified bsDMARD prescription, higher age at initiation, early line of treatment, citrate presence, longer disease duration, prescription by an attached practitioner, and male sex, as associated with treatment retention. Among these results, superior retention of bsDMARDs compared to boDMARDs was the main finding of this study. An analysis of each molecule separately found that superior retention of bsDMARDs was only observed with ETN. This is concordant with a recent Swedish study focused on the initiation of either biosimilar of ADA and ETN, which demonstrated a hazard ratio of treatment retention at 1 year in favor of SB4 biosimilar of ETN, while no differences had been found between HUMIRA and its biosimilars (16). For ADA, another study did not find significant differences between bsDMARD and boDMARD at initiation (41). The difference between molecules in our study is not a consequence of differential retention of each molecule since no difference was observed comparing ADA and ETN. However, the number of patients under ADA was half the number of patients under ETN. The equivalence of these two molecules in terms of retention is a well-known fact either in RA (42), SpA (43), or PsA (44). It is noteworthy that nearly all previously cited studies focused on the switch instead of the initiation of bsDMARDs. An Italian study found opposite results with much better retention of boDMARDs over bsDMARDs at initiation (25). Since this last letter included patients treated with intravenous infliximab and intravenous rituximab, the results are not exactly comparable to the one in our study.

In patients with RA, bsDMARDs had significantly better retention compared to boDMARDs, while it was not the case in PsA and SpA. In a study comparing HYRIMOZ^®^ to IMRALDI^®^, differences in retention between these two biosimilars were significant only in RA when analyzing retention according to the disease (45). No study with potential explicative factors for this difference between diseases had been found in the literature.

When used as the first biologic, bsDMARDs and boDMARDs exhibited no significant differences in terms of retention, even if the median of retention was higher for bsDMARDs. This is concordant with the results of Di Gisueppe et al., with no difference in the retention of treatment for ADA, while there was a slightly better retention of biosimilar ETN (16). When used as second-line bDMARDs, bsDMARDs demonstrated better retention compared to boDMARDs, while there were no differences for the third line of prescription. To our knowledge, this is the first report of such an effect of the line of treatment in differential retention of bsDMARDs and boDMARDs. Considering the line of prescription as a parameter for the predictive factor of treatment retention, the literature is in accordance with this finding. In an Australian study about patients with RA, a decrease in persistence rates with the line of treatment is described (46). The same results with better retention of TNF inhibitors in biologically naïve compared to first or second switchers in RA are found in the CORRONA registry (47) in line with results of the ANSWER study (48). In PsA and SpA, it has been demonstrated that the retention of treatment was lower as patients already experienced more DMARDs (49, 50). A recent meta-analysis of drug persistence in SpA found higher retention for first-line bDMARDs compared with further lines (51). The same finding is described in psoriatic arthritis (52).

Prescription by an attached practitioner was associated with better retention of treatment in general and for boDMARDs. To our knowledge, this point is nearly never addressed in clinical studies, most of them using only hospital-based databases. Literature about the physician–patient relationship in the context of rheumatic disease is also scarce. However, it has been shown that patients are in demand of the availability of physicians. Appointment delays, lack of continuity of care, or feeling of a lack of interest from the physician are described as negative factors in the relationship with a possible impact on disease and effect of treatment (53, 54). A hypothesis could be that rheumatologists with both a hospital and office-based practice are easily available with patients having a longer and deeper trust in them.

Better retention of treatment in men is a well-known feature in the literature. A recent study about the retention of bDMARDs in the same three rheumatic diseases as here found that female gender was associated with more cessation of treatment, but this was not significant in the multivariable analysis (55). This phenomenon is well-known in SpA (56) in which women have worse patient-reported outcomes as demonstrated recently in the CORONA registry (57). In a study about RA, PsA, and SpA, the results are also concordant with more cessation of treatment in women (58).

Citrated treatments display superior retention compared to citrate-free treatments, either if considering all prescriptions or only bsDMARD prescriptions. This result was unexpected. Indeed, citrate buffer-induced pain at the injection site is a long-time known element with such an effect demonstrated for epoetin alfa injection in 1998 (59) or in a randomized controlled trial about growth hormone injection in 2006 (60). For bDMARDs, such a negative impact has also been shown (61, 62). In terms of citrate, sodium citrate and monohydrated citric acid are the two subtypes of this excipient. This led to the development of citrate-free drugs, including the formula modification of HUMIRA with a demonstration of a better persistence of citrate-free HUMIRA compared to the citrated one (62). Citrate presence is not significant in the multivariable analysis of predictive factors for the cessation of treatment, probably due to still unknown confounding factors. Moreover, in a switch study comparing two citrated biosimilars of ADA, namely SB5 and GP2017, the difference in favor of GP2017 was observed (45). Even if both are citrated, there are small differences in terms of the citrate-buffer subtype with SB5 containing sodium citrate and monohydrated citric acid, while GP2017 only contains the latter.

Previous studies demonstrated the variable impact of disease duration on the retention of treatment with some demonstrating better retention in case of longer disease duration (63), while others did not find such an impact (55).

Looking at predictive factors of retention, the multivariable analysis found that longer disease duration, bsDMARD prescription, attached practitioner prescription, early line of treatment, and male sex were associated with longer retention, while long disease duration and early line of treatment were found for bsDMARDs and only attached practitioner prescription for boDMARDs.

The results of the analysis of side effects, particularly infections are quite surprising. Indeed, bsDMARDs are considered biologically similar to boDMARDs, thereby implying that the safety profile should be equivalent. In the literature, studies comparing the safety of bsDMARDs to boDMARDs did not find any difference in terms of adverse events, whatever the molecule (64–68). Our hypothesis is that boDMARDs are more often prescribed in more frail patients, who are intrinsically more prone to adverse events, because of rheumatologists’ greater experience of these treatments.

Our study has some limitations. Due to its retrospective nature, there were missing data, especially for disease activity, which was available only for 31.8% of patients, which did not allow us to analyze this point with precision. No sufficient data were available to evaluate comorbidities of patients, which is a known factor that impacts retention of treatments (69–71) and may be an explanation for some of our results. Another limit is the exclusion of patients who were under boDMARDs switched to bsDMARDs which could have limited the conclusions we could draw from the results. However, this pitfall was impossible to avoid taking into account that the objective of this study was focused on initiation. Despite those limits, this study has some strengths. It encompasses the prescriptions of bDMARDs from both ambulatory practice and hospital prescriptions, which is rarely the case. Patients with RA, PsA, and SpA were included. Despite the retrospective, and not so randomized, nature of the study, groups were nearly comparable for all studied characteristics points. It is also one of the few studies focused on comparing the retention of boDMARDs and bsDMARDs in the context of the initiation of treatment.

To conclude, despite the amount of knowledge supporting the efficacy and safety of bsDMARDs, their systematic prescription to initiate a new molecule is not a reflex among French rheumatologists. The maintenance of bsDMARDs is superior to boDMARDs, particularly for ETN, and in the context of RA. Citrate impact on the retention of treatment seems to still be full of mystery, which needs further studies to clarify its impact. bDMARD prescription in a long-standing disease as early line prescription is associated with better retention, as well as a prescription by a not fully hospital-based rheumatologist, probably reflecting the importance of a close and trusting relationship between patients and physicians. Future studies are needed to confirm those results while taking into account disease activity and comorbidities to assess with more precision underlying features of the treatment retention.

## Data availability statement

The raw data supporting the conclusions of this article will be made available by the authors, without undue reservation.

## Ethics statement

The studies involving human participants were reviewed and approved by Poitiers Local Ethic Committee. Written informed consent for participation was not required for this study in accordance with the national legislation and the institutional requirements.

## Author contributions

GB and EG: conceptualization. GL and AD: formal analysis and writing – original draft. GL, GB, AD, PC, VG, MG, LM, FM, EV, EH, J-HS, R-MF, and EG: investigation. GL and EG: methodology and writing – review and editing. EG: supervision. All authors contributed to the article and approved the submitted version.
